# Enhanced biofilm prevention activity of a SPLUNC1-derived antimicrobial peptide against *Staphylococcus aureus*

**DOI:** 10.1371/journal.pone.0203621

**Published:** 2018-09-14

**Authors:** Zhongjie Yu, Berthony Deslouches, William G. Walton, Matthew R. Redinbo, Y. Peter Di

**Affiliations:** 1 Department of Environmental and Occupational Health, University of Pittsburgh, Pittsburgh, PA, United States of America; 2 Center for Molecular Genetics, Institute for Translational Medicine, Qingdao University, Qingdao, China; 3 Department of Microbiology and Molecular Genetics, University of Pittsburgh, Pittsburgh, PA, United States of America; 4 Departments of Chemistry, Biochemistry, and Microbiology, University of North Carolina, Chapel Hill, NC, United States of America; Nanyang Technological University, SINGAPORE

## Abstract

SPLUNC1 is a multifunctional protein of the airway with antimicrobial properties. We previously reported that it displayed antibiofilm activities against *P*. *aeruginosa*. The goal of this study was to determine whether (1) the antibiofilm property is broad (including *S*. *aureus*, another prevalent organism in cystic fibrosis); (2) the α4 region is responsible for such activity; and (3), if so, this motif could be structurally optimized as an antimicrobial peptide with enhanced activities. We used *S*. *aureus* biofilm-prevention assays to determine bacterial biomass in the presence of SPLUNC1 and SPLUNC1Δα4 recombinant proteins, or SPLUNC1-derived peptides (α4 and α4M1), using the well-established crystal-violet biofilm detection assay. The SPLUNC1Δα4 showed markedly reduced biofilm prevention compared to the parent protein. Surprisingly, the 30-residue long α4 motif alone demonstrated minimal biofilm prevention activities. However, structural optimization of the α4 motif resulted in a modified peptide (α4M1) with significantly enhanced antibiofilm properties against methicillin–sensitive (MSSA) and–resistant (MRSA) S. *aureus*, including six different clinical strains of MRSA and the well-known USA300. Hemolytic activity was undetectable at up to 100μM for the peptides. The data warrant further investigation of α4-derived AMPs to explore the potential application of antimicrobial peptides to combat bacterial biofilm-related infections.

## Introduction

Human SPLUNC1 (short palate lung and nasal epithelial clone 1) is a 256-amino acid multifunctional protein of the innate immunity secreted in the human respiratory tract. It binds to lipopolysaccharide (LPS) and exerts bacteriostatic as well as antibiofilm effects[1–4]. In addition, it acts as a fluid-spreading surfactant, which facilitates mucus clearance[5–7]. SPLUNC1 has several alternative names. It is referred to as BPIFA1 (BPI fold containing family member A1) because of its structural similarity to bacterial permeability increasing protein (BPI)[5], lung-specific protein X or LUNX[2], or SPURT (secreted protein from upper respiratory tract)[2, 8]. We will refer to it as SPLUNC1 in this report.

The air is a nonsterile environment[9–13]. Therefore, the human airway is continuously exposed to potential pathogens[14]. Yet, infections are relatively rare. The airway is equipped with a mucociliary apparatus (MCA)[15], which is largely responsible for protecting the host through mucociliary clearance of microbial organisms. An important component of the MCA is the airway surface liquid (ASL) lining the airway and acting as a lubricant for normal ciliary function[6, 16–18]. In addition, the ASL contains a variety of antimicrobial factors including proteins and short peptides known as antimicrobial peptides (AMPs)[19–24]. SPLUNC1 helps regulate the ASL by providing a mechanism for controlling Na+ absorption through the inhibition of the epithelial sodium channel, ENaC[25–27]. In addition, direct antimicrobial activity of SPLUNC1 has been observed[2, 3, 5–7, 28].

Multiple domains within the SPLUNC1 secondary structure have been previously elucidated[7, 28]. One particular motif, called α4, displays a helical structure. On closer examination, this domain appears to exhibit a cationic amphipathic structure similar to that of well-known natural AMPs[29–32], with a positive charge of 2. We hypothesized that the antimicrobial α4 motif of SPLUNC1 with the characteristic of natural antimicrobial peptides can be used as a novel standalone antibiofilm agent. We report herein the impact of the α4 motif on the antibacterial properties of SPLUNC1 and the enhanced antibiofilm properties of the α4 region based on structural optimization.

## Materials and methods

### Protein and peptide synthesis

The recombinant proteins SPLUNC1 and SPLUNC1Δα4 (Wingtip) were expressed and purified as previously described[7]. Colistin sulfate was purchased from Sigma (St. Louis, Mo, USA). Synthetic α4 (ILKPGGGTSGGLLGGLLGKVTSVIPGLNNI), α4M1 (ILKKWWGTSGGLLGGLLGKVTSVIKGLNNI), and our control peptide for mammalian toxicity WLBU2 (RRWVRRVRRVWRRVVRVVRRWVRR) were synthesized using standard Fmoc (9-fluorenylmethoxy carbonyl) synthesis protocols as previously described[33] and purification achieved by reversed-phase high-pressure liquid chromatography on Vydac C18 or C4 columns (The Separations Group). The identity of each peptide was established by MS (Electrospray Quatro II triple quadrupole mass spectrometer).

### Bacteria

All methicillin-resistant *S*. *aureus strains* are clinical isolates anonymously provided by Cystic Fibrosis Foundation and the medical laboratory of the University of Pittsburgh Medical Center. These strains have been used in previous studies, and the names are SA 0150–10, SA 0467–1, SA 0122–12, SA 0193–12, SA 0092–19, and SA 0187 in addition to the well-known USA300[34]. There was only one methicillin-sensitive *S*. *aureus* (MSSA) strain, and it was purchased from ATCC (ATCC49775).

### Biofilm assay

We used a slightly modified version of the microtiter plate assay as previously described[5]. Briefly, log-phase bacteria were diluted in DMEM (to facilitate biofilm formation, as previously reported[35, 36]) to 10^8^ CFU/mL based on pre-determined bacterial numbers that correlate with the optical density readings using a spectrophotometer. A 50μL volume of protein or peptide (in PBS), at different concentrations, was added to 50μL of bacterial suspension in a sterile 96-well polystyrene plate. The final bacterial concentration of the mixture is 5x10^7^ CFU/mL, 50-fold compared to 10^6^ CFU/mL in standard planktonic growth inhibition assays for adequate bacterial attachment, as required for biofilm formation. After 24h hours[37, 38] (at every 6h intervals for kinetic of biofilm formation assay) of bacterial biofilm growth at 37°C (no shaking), the supernatant was discarded. The plate was washed with PBS prior to staining with 125μL of 0.5% Crystal Violet (in 20% Ethanol) for 15 minutes. Excess stain was removed by washing twice with distilled water[6]. Crystal violet-stained biomass was dissolved in 150μL of 95% ethanol and measured using a plate reader at 620nm. Untreated bacteria (100% bacterial attachment), served as positive control. Wells with a mixture of sterile DMEM and PBS were used to control for possible contamination. Biomass in different treatment groups was quantified as percent OD of the positive controls.

### Red blood cell lysis assay

Hemolytic assays were performed using red blood cells (RBCs) isolated from heparinized human blood obtained anonymously from the Central Blood Bank of Pittsburgh. The erythrocytes were separated by Histopaque gradient centrifugation and then resuspended to 2% (vol/vol) in PBS, as previously described[39]. To determine RBC lysis, a volume of 50 μl (1:4) of the RBC suspension was mixed with peptides at variable concentrations ranging from 0 to 100μM to a total volume of 200μl in a round-bottom 96-well plate. The reaction mixture was incubated at 37°C for 60 min with gentle shaking. To analyze the RBC lysis, the RBC-peptide mixture was spun at 600*g* for 5 min, and 80μl of the supernatant transferred to 120μl (1:2.5) of RBC lysis buffer (final dilution 1:10) in a flat-bottom 96-well plate for spectrophotometric analysis. Similarly, 0 to 50μl of untreated RBCs was diluted in RBC lysis buffer to a final volume of 500μl (up to 1:10 dilution), and the hemoglobin suspensions were used to produce a standard RBC lysis curve. The average absorbance values of the supernatants of all samples (200μl) in triplicates were measured by using a microplate reader at 550 nm as an indicator of hemoglobin released from lysed cells. These experiments were verified by three independent trials.

### Statistical analysis

Data generated were analyzed as indicated in each figure legend by one-way or two-way ANOVA using multiple comparisons test, depending on the data set. These analyses were performed using GraphPad Prizm software.

## Results

### Deletion of the α4 domain reduces SPLUNC1 activity

**Fig 1:**
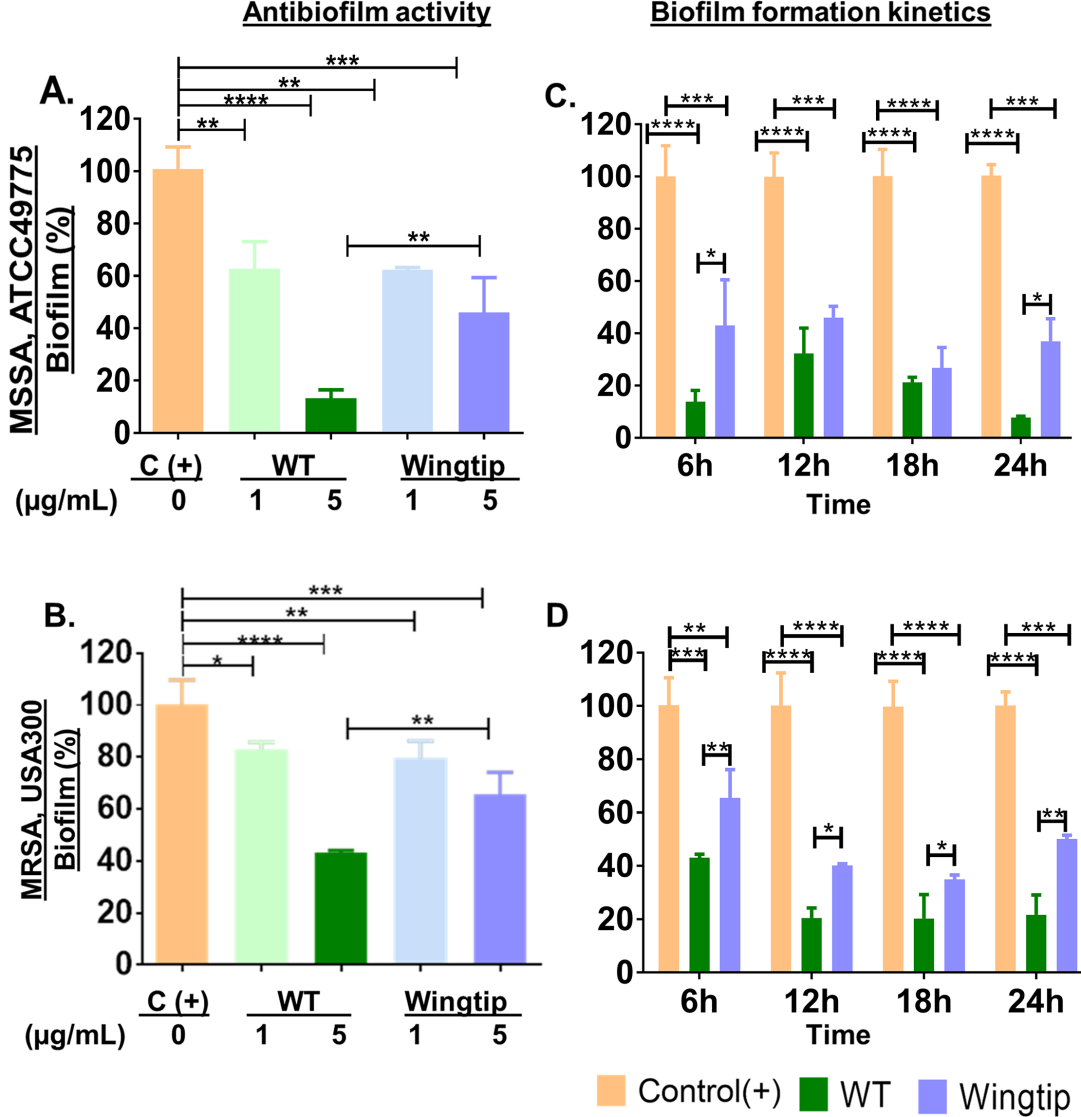
Dependence of SPLUNC 1 antibiofilm prevention activity on the α4 domain. SPLUNC1 displayed higher reduction in *S*. *aureus* biofilm than Δα4; MSSA, ATCC49775: **A,** antibiofilm assay and **C,** biofilm growth inhibition kinetics; MRSA USA300; **B,** antibiofilm assay and **D,** biofilm growth kinetics. *denotes statistical significance at *P<0.05 using one-way ANOVA by Tukey’s multiple comparisons test (A and B) or multiple t tests (C and D); **P<0.005; ***P<0.001; ****P<0.0001.

To examine the role of the α4 region in SPLUNC1 antibiofilm properties, we first compared SPLUNC1 (WT) and the Δα4 protein (Wingtip) for antibiofilm prevention activities against both methicillin-sensitive (MSSA, ATCC49775) and methicillin-resistant *S*. *aureus* (MRSA, USA300) (Fig 1A and 1B). Bacteria (MSSA and MRSA), treated with the mutant protein (Wingtip, 5μg/mL), displayed 43% and 65.5% of biofilm formation, respectively compared to 13.8% and 43% biofilm mass by the same bacterial strains treated with SPLUNC1-WT. Lower activity was observed at protein concentration of 1μg/mL compared to 5μg/mL, as expected. These results indicate a 1.5- (MRSA) to 3.1-fold (MSSA) reduction in activity when the α4 region is deleted from the WT protein. Next, we used the effective concentration of 5μg/mL for examination of biofilm growth inhibition kinetic in the presence of SPLUNC1 or the Δα4 protein (Fig 1C and 1D). The WT protein demonstrated a lower biofilm mass by 6h, 17% for MSSA and 43% for the MRSA strain compared 43% (MSSA) and 65.5% (MRSA) against these organisms for the Δα4 protein. The highest ativity was achieved at 24h with 7.7% for MSSA and 21.7% for MRSA of detectable biomass for the WT protein compared to 37% for MSSA and for 50% MRSA biofilm for Δα4-treated bacteria at 24h (Fig 1C and 1D). This is a 2 to 5-fold higher activity for the WT protein compared to ΔA4, always with statistical significance (P<0.05 to 0.001).

### Antimicrobial properties of synthetic α4 motif can be enhanced by sequence optimization

**Fig 2:**
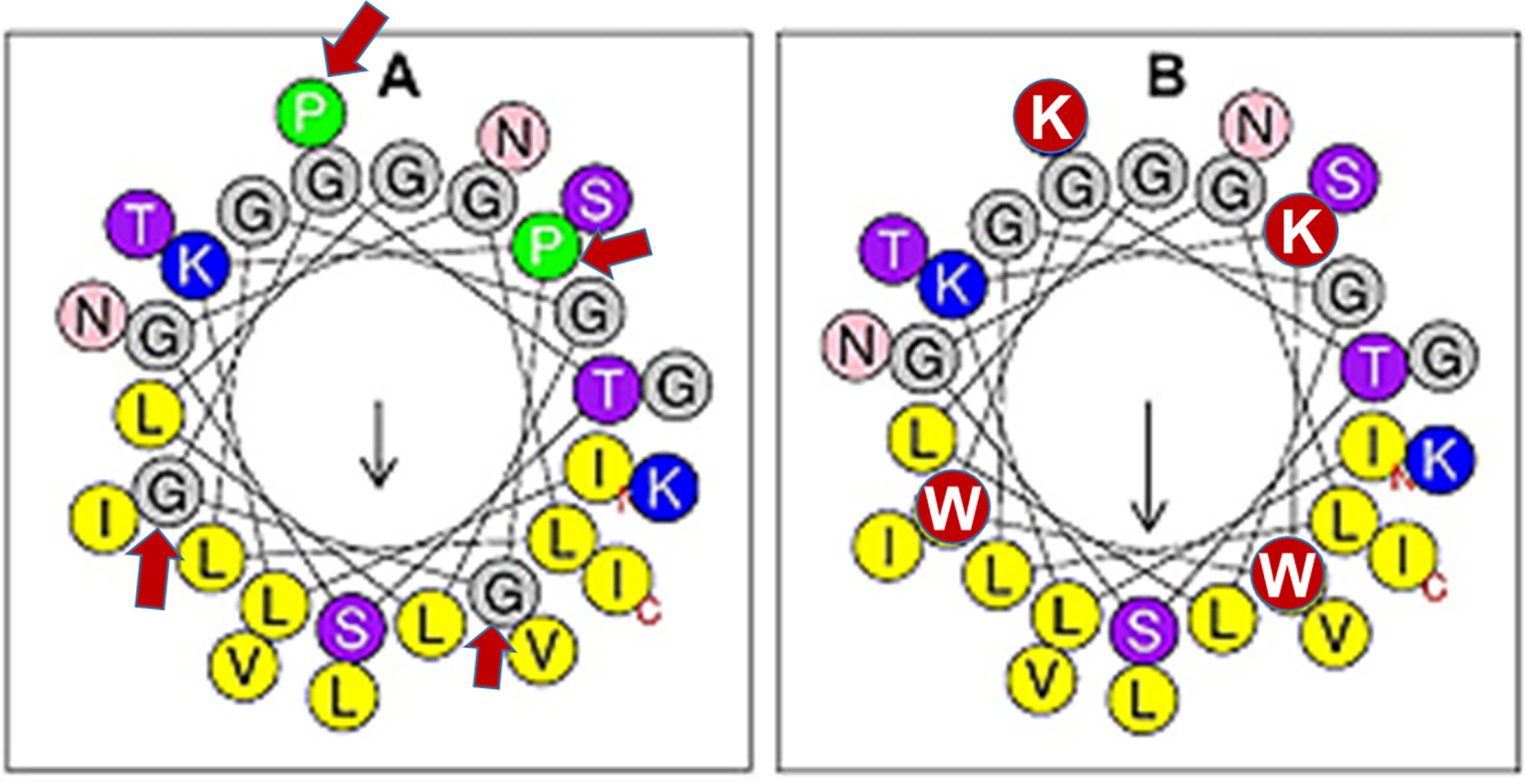
**Helical wheel diagrams of (A) α4 and (B) α4M1.** Black arrows indicate the direction of the hydrophobic moments (left, μH = 0.373; right, μH = 0.563 (http://heliquest.ipmc.cnrs.fr/), and red arrows in (A) indicate the sites of mutagenesis. Hydrophobic amino acids are in yellow and cationic in blue, except for the red circles denoting (B) amino acid substitutions.

Because the antibiofilm activities of SPLUNC1 was affected by the deletion of the α4 helical domain, we performed a helical wheel analysis (Fig 2) on the α4 region (A) and observed an amphipathic structure similar to that of classical AMPs. However, the amphipathicity, as measured by the hydrophobic moment (μH = 0.373), is minimal due to a positive charge of only 2. Considering the importance of the membrane perturbation properties of AMPs in overcoming multidrug resistance and biofilm formation by bacterial pathogens, we sought to enhance the amphipathicity of α4 by doubling the positive charge using Lys (K) to replace the two Pro (P) residues on the hydrophilic side. In addition, because there are two Gly (G) residues in the hydrophobic region, we used the membrane interfacial-seeking amino acid Trp (W) to replace these two residues, resulting in the helical wheel structure in Fig 2B. These changes led to a 51% increase in the hydrophobic moment, a measure of the amphipathicity (α4M1 μH = 0.563).

**Fig 3:**
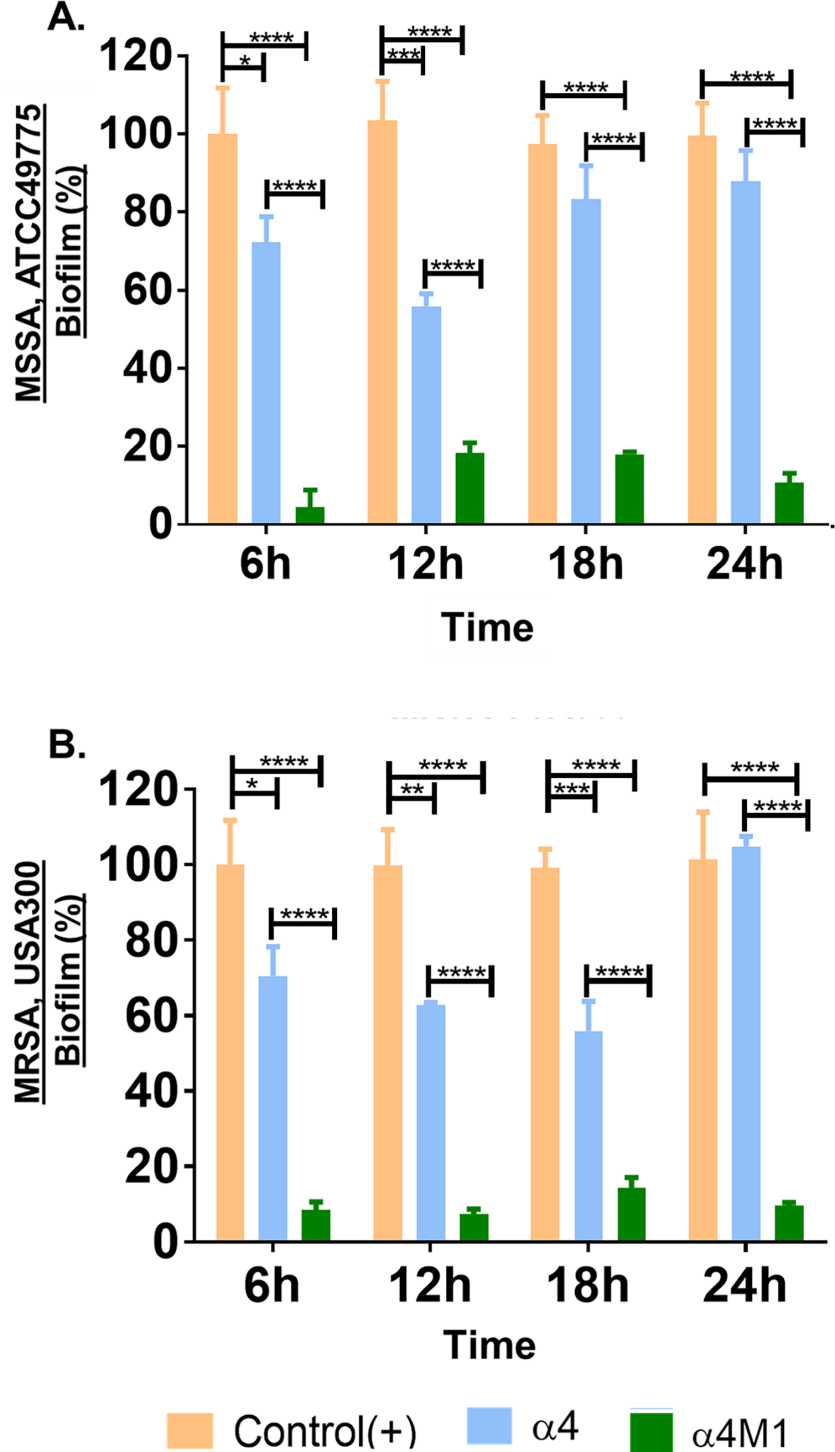
The synthetic α4 domain can be structural optimized for enhanced biofilm prevention properties. The α4 sequence was compared to α4M1 for biofilm inhibition kinetic activities against MSSA ATCC49775 (A) and MRSA USA300 (B); P values (*P<0.05, **P<0.005, ***P<0.001), ****P<0.0001 were determined by multiple t tests.

Interestingly, the synthetic α4 domain alone (32μM) demonstrates lower biofilm prevention activity against both MSSA and MRSA (Fig 3) compared to the WT protein shown in Fig 1, which indicates that this region (although important) alone is not accounted for the entire antimicrobial function of this protein. Structural optimization, however, was sufficient to overcome the lack of strong activity of α4. As shown by the biofilm prevention kinetics in Fig 3, the α4-derived α4M1 was able to prevent *S*. *aureus* (MSSA ATCC49775 and MRSA USA300) biofilm by 80–90%, from 6h to 24h of biofilm growth.

**Fig 4:**
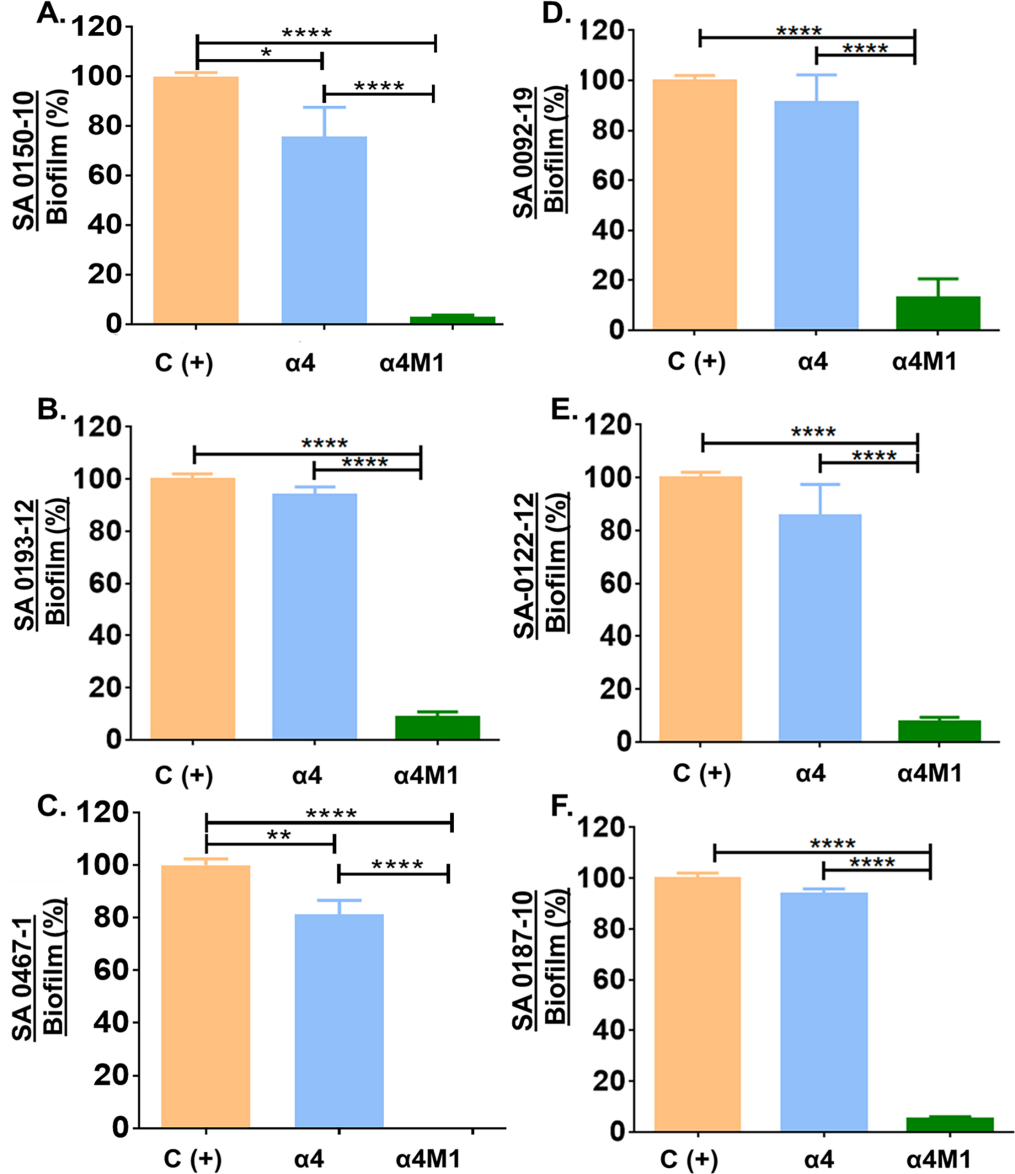
**Biofilm prevention activities of α4 and α4M1 using six clinical strains of methicillin-resistant *S*. *aureus* (A through F).** Bacterial biofilm were measured by crystal violet 24h after bacterial incubation at 37°C in the presence or absence (control) of the indicated peptides; P values (*P<0.05, **P<0.005, ***P<0.001, ****P<0.0001) were determined by one-way ANOVA using Bonferroni’s multiple comparisons test.

To test whether the observed activity is not strain-specific, we further compared α4 and α4M1 for antibiofilm prevention activities against six additional clinical strains of MRSA (Fig 4). The derived peptide α4M1 retained antibiofilm prevention activities against those strains, with a reduction in biofilm formation by 80–99%, compared to a modest 5–20% reduction in biofilm mass by the parent peptide α4.

**Fig 5:**
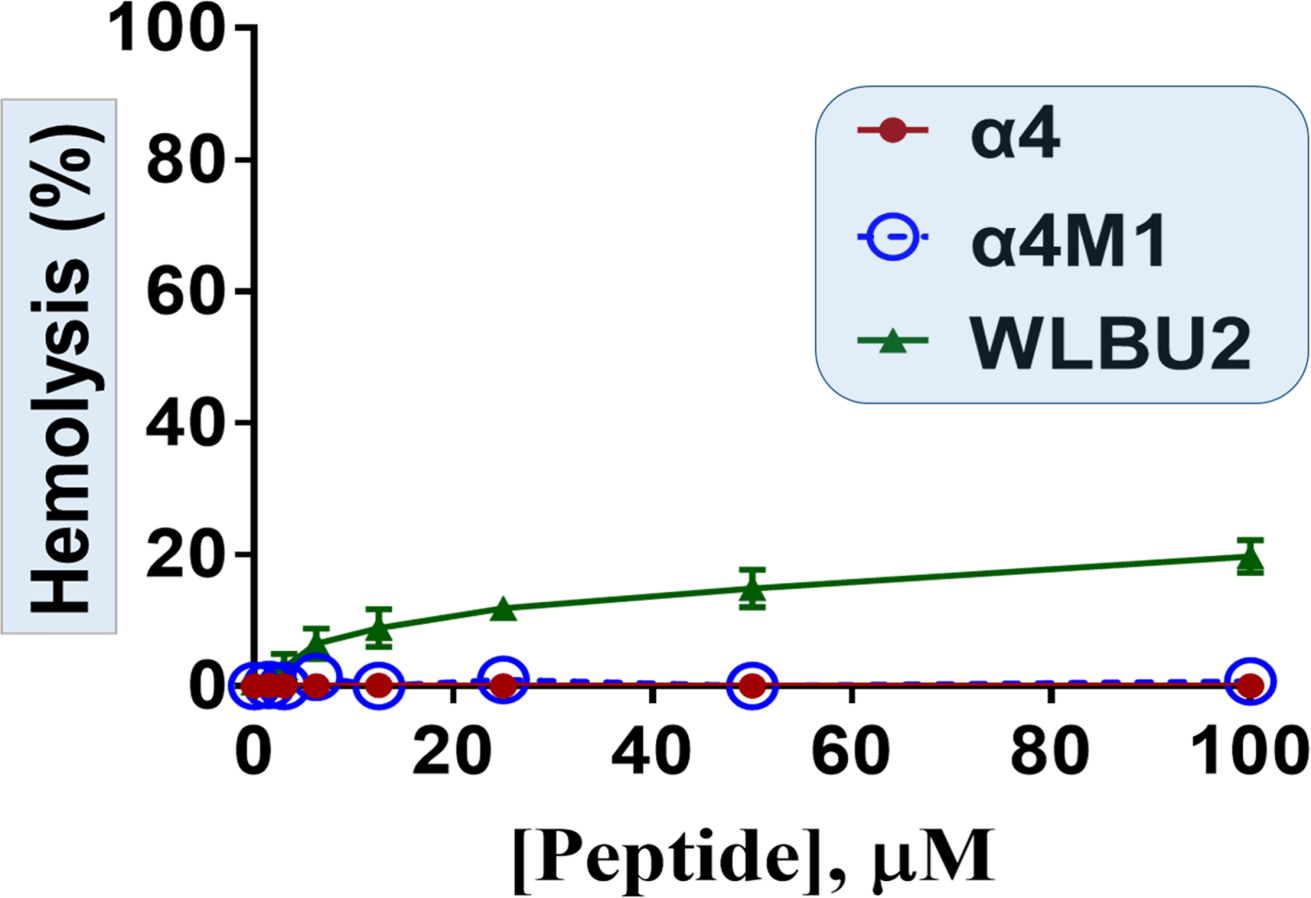
Negligible hemolytic activity of α4 and α4M1, compared to the engineered AMP WLBU2. Freshly isolated human erythrocytes in PBS were incubated with each peptide at the indicated concentrations for 1h and percent hemolysis determined according to Materials and Methods.

As a primary characterization of the cytotoxic property, we compared the two peptides for hemolytic activities using freshly isolated human erythrocytes. Both peptides show no detectable hemolytic activity at concentrations up to 100μM (Fig 5), in contrast to the engineered AMP control WLBU2[31, 33, 40, 41], which displayed up to 20% hemolysis.

## Discussion

Biofilms are highly resistant to clinical treatment by traditional antibiotics[42, 43] and are an important aspect of the pathogenicity of bacterial pathogens associated with respiratory infections, particularly in chronic disease such as cystic fibrosis[44, 45]. The host relies on the MCA to clear most potential pathogens from the airway. The MCA includes the ASL as an important component that facilitates mucociliary clearance. ASL consists of surfactants, antimicrobial molecules such as SPLUNC1, typical cationic AMPs, in addition to many other molecules (immunoglobulins, proteases, etc.). Hence, mucociliary clearance occurs by a combination of mechanisms, which prevent microbial attachment to and colonization of the airway epithelium[46–48]. We previously reported that SPLUNC1 displayed activity against *P*. *aeruginosa* biofilm[28]. The premise of this study is that SPLUNC1 displays broad activity and, therefore, would prevent biofilm growth by gram-positive (e.g. *S*. *aureus*) organisms as well as gram-negative (e.g. *P*. *aeruginosa*) bacteria[2, 28]. Importantly, the cationic amphipathic motif α4[7, 28] can be optimized as a standalone antibiofilm peptide. In this study, we demonstrated that SPLUNC1 was also able to prevent *S*. *aureus* biofilm, in contrast to a previous report[7]. In addition, this activity was largely dependent on the α4 motif, which was optimized successfully for enhanced antibiofilm properties.

The α4 region[7] of SPLUNC1 has a helical amphipathic structure with a sequence of 30 amino acid residues long and a high content of hydrophobic residues (hydrophobicity H = 0.558, Fig 2). As shown by the helical wheel analysis, the amphipathicity is minimal (μH = 0.373) in α4, reflective of a weak hydrophilic motif (charge +2) and minimal antibiofilm activity. Hence, the changes made in the parent α4 structure were intended to modestly increase the cationic content (charge = +4) and the amphipathicity (μH = 0.563). While the antibiofilm mechanism of SPLUNC1 is not entirely clear, it does not appear to be affected by the type of bacterial organism (gram negative vs. gram positive), as shown by the antibiofilm activity against both MSSA and MRSA strains. This activity is similar to the broad-spectrum activity of AMPs. Considering that the short α4 sequence is derived from a natural protein in human, we thought that the minor structural modifications might not affect cytotoxicity toward mammalian cells. One of the concerns with the structural optimization of natural AMP sequences is that enhancing the antibacterial potency may also result in increased toxicity toward mammalian cells. To illustrate, we compared the hemolytic profile of the engineered AMP WLBU2, which is in advanced preclinical development, with that of α4 and α4M1. WLBU2 has been extensively characterized both *in vitro* and *in vivo[30, 33–35, 40, 41, 49–52]*. It displays broad-spectrum activity against the most common MDR bacteria known as ESKAPE pathogens[34] and outperforms the last-resort antibiotic colistin against these MDR clinical strains. Hence, Lessons learned from these extensive studies led us to consider only two Trp residues in the hydrophobic face. Consequently, both α4 and α4M1 demonstrate no detectable hemolytic activities while the antibiofilm properties of the structurally optimized α4M1 are markedly enhanced.

The data suggest that the higher cationic content is highly relevant to the enhanced antibiofilm property of α4M1 possibly by interfering with bacterial attachment to solid surfaces, whereas the inclusion of Trp in the hydrophobic motif most certainly plays a role in overall activity. As future direction, a logical step would be to explore whether bacterial membrane-AMP electrostatic interactions may interfere with bacterial attachment, the first principal step in biofilm formation. Although beyond the scope of the current studies, we plan to explore biofilm prevention by anti-dispersion activity and specific applications to biofilm-related infections in future studies.

## Conclusions

While our initial report seems to suggest a lack of activity of SPLUNC1 against *S*. *aureus*, this lack of activity appears to be limited to one strain[7]. SPLUNC1 and its derived AMPs displayed antibiofilm prevention activities against multiple strains of *S*. *aureus* (both MSSA and MRSA including six additional clinical MRSA strains[34]). This activity appears to depend at least partly on the α4 motif and can be enhanced by increasing the cationic and Trp content toward enhancing the amphipathicity as well as the hydrophobicity. Such a modest modification does not increase hemolytic or cytotoxic activity of the α4-derived peptide but noticeably increased the antibiofilm activity against both MSSA and MRSA. The data warrant further investigation of α4-derived AMPs to explore the potential application of AMPs to bacterial biofilm-related infections such as those associated with surgical sites, wound, or cystic fibrosis.

## Supporting information

S1 Fig (XLSX)
